# Decreased temperature variance associated with biotic composition enhances coastal shrub encroachment

**DOI:** 10.1038/s41598-020-65161-3

**Published:** 2020-05-19

**Authors:** Lauren K. Wood, Spencer Hays, Julie C. Zinnert

**Affiliations:** 1 Department of Biology, Virginia Commonwealth University, 1000 West Cary St, Richmond, VA 23225 USA; 2 Integrative Life Sciences Doctoral Program, Virginia Commonwealth University, 1000 West Cary St, Richmond, VA 23225 USA; 3 Department of Statistics, Indiana University, 919 E. 10th St, Bloomington, IN 47408 USA

**Keywords:** Ecophysiology, Ecosystem ecology

## Abstract

Regime shift from grasslands to shrub-dominated landscapes occur worldwide driven by altered land-use and climate change, affecting landscape function, biodiversity, and productivity. Warming winter temperatures are a main driver of expansion of the native, evergreen shrub, *Morella cerifera*, in coastal landscapes. Shrub establishment in these habitats alters microclimate, but little is known about seasonal differences and microclimate variance. We assessed influence of shrubs on microclimate variance, community composition, and community physiological functioning across three vegetation zones: grass, transitional, and shrub in a coastal grassland. Using a novel application of a time-series analysis, we interpret microclimatic variance modification and elucidate mechanisms of shrub encroachment at the Virginia Coast Reserve, Long-Term Ecological Research site. As shrub thickets form, diversity is reduced with little grass/forb cover, while transpiration and annual productivity increase. Shrub thickets significantly reduced temperature variance with a positive influence of one day on the next in maximum air, minimum air, and maximum ground temperature. We also show that microclimatic temperature moderation reduces summer extreme temperatures in transition areas, even before coalescence into full thickets. Encroachment of *Morella cerifera* on the Virginia barrier islands is driven by reduced local exposure to cold temperatures and enhanced by abiotic microclimatic modification and biotic physiological functioning. This shift in plant community composition from grassland to shrub thicket alters the role of barrier islands in productivity and can have impacts on the natural resilience of the islands.

## Introduction

Woody vegetation is rapidly encroaching into grassland globally as a result of altered environmental drivers^1–3^. The abrupt conversion to shrub-dominated habitats includes drivers such as altered fire or grazing pressures, increased atmospheric CO_2_, and altered temperature ranges^3–6^. Warming temperatures associated with macroclimatic change, particularly in winter minima, can expand the range limits of cold-intolerant shrub species^3,6–8^ while macroclimatic variance in precipitation enhances success of woody vegetation, particularly in the transition ecotones^9,10^. Thus, no single factor provides dominant influence on woody encroachment.

Increased annual temperatures may improve physiological functioning of encroaching species, possibly as a feedback between abiotic microclimatic conditions and shrub success to promote range expansion for woody shrubs^3,7^. On smaller scales, encroaching woody vegetation frequently modifies microclimatic conditions resulting in intra- and interspecific facilitation^11–14^. Changes in microclimatic temperatures within shrub thickets include temperature moderation with diminished instances of extreme temperatures that can decrease productivity and cause physiological damage of xylem structures^3,6^. In areas with warming winter temperatures, thicket-forming species that are vulnerable to cold-associated damage, such as freezing-induced embolism, are able to take advantage of fewer cold temperature extremes^1,6,15^. Multiple encroaching species are cold intolerant, thus a warming climate and self-reinforcing microclimatic modification allow aggressive expansion of shrubs in to historical grassland^3,8,11,13,16–18^.

Regime shifts in vegetation and the resulting modified microclimate beget community changes, often leading to increased diversity with sparse canopy shrubs and decreased diversity in densely leaved canopies^13,18,19^. These altered plant communities tend to have higher productivity and carbon assimilation and may support different faunal species. Changes in productivity increase soil nutrient assimilation and alters soil microbial communities^20^. While the drivers and stresses differ among various habitats undergoing woody encroachment, resulting community change and ecosystem consequences are similar.

Among landscapes that have experienced regime shift from grass to woody vegetation are low elevation swale/slack communities along the United States mid-Atlantic and Gulf coasts^21–26^. Coastal ecosystems in this region have reduced diversity relative to inland communities^27^ with unique environmental stressors. On a large scale, wind, waves, tides, and storms result in dynamic sediment movement, sea spray, low canopy cover, and highly reflective sandy sediment, which increase irradiance. Regionally, atmospheric and oceanic climate change drivers are altering temperatures, storm regime, and sea level^25,28^. Species diversity is restricted as a result of these environmental filters^27,29^. For island landscapes, species diversity is additionally filtered by dispersal^26,27,30^. The vegetation on barrier islands is further limited by the availability of freshwater and precipitation regime; barrier islands have a freshwater lens that is entirely precipitation-fed^21,31,32^. Coastal habitats are understudied environments where vegetation dynamics present an unique opportunity to understand shrub encroachment mechanisms.

**Figure 1:**
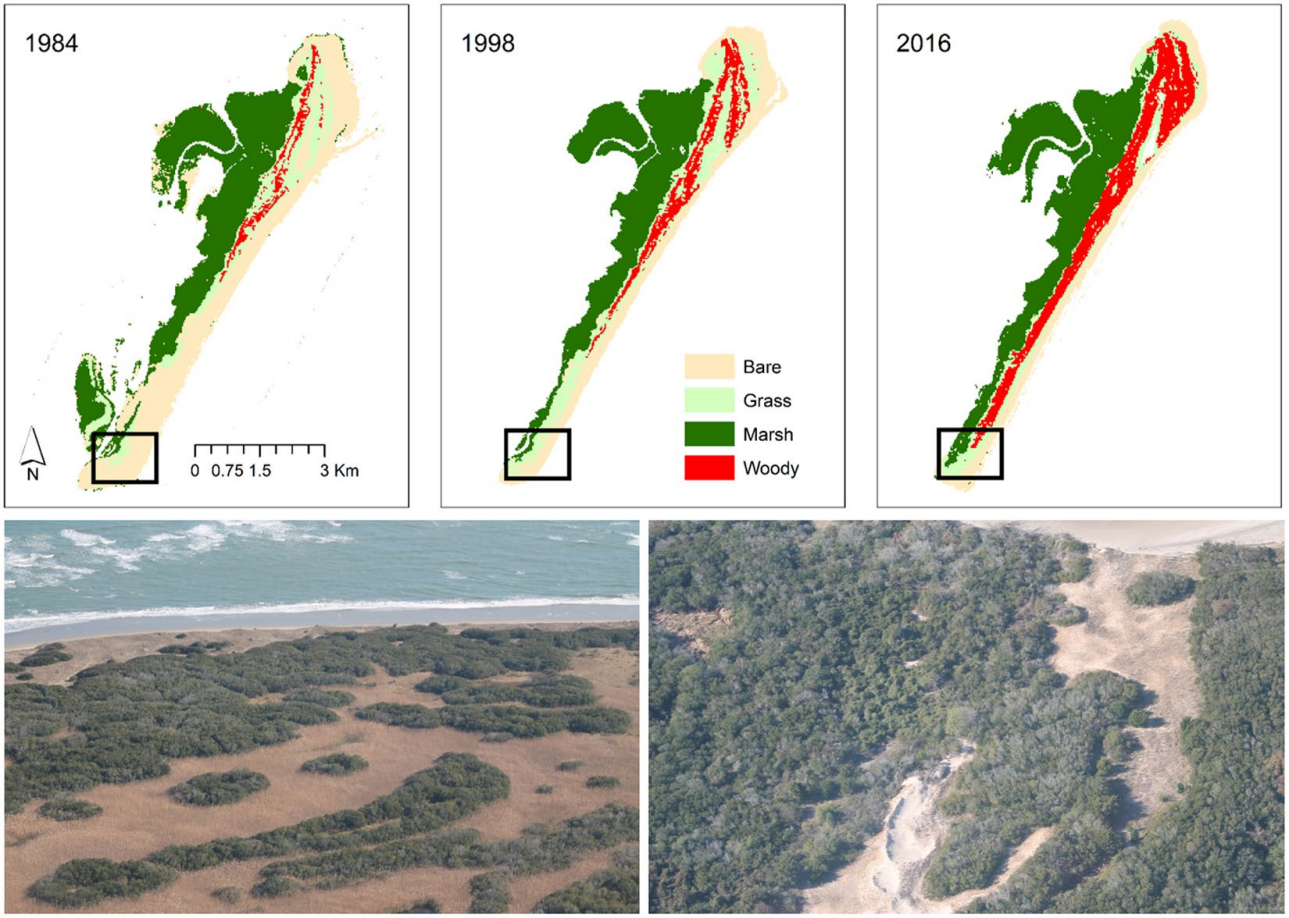
Shrub cover has expanded on Hog Island, Virginia since 1984 represented in red. Data are from Zinnert *et al*.^33^. The black box denotes the study area. Photos of shrub cover on the southern portion of the island from November 2017 provided by Julie C. Zinnert.

Over the past 32 years, shrub cover has increased on the Virginia barrier islands by ~40% despite an overall decrease in island area due to sea level rise and erosion (Fig. 1)^28,33^. *Morella cerifera* (wax myrtle), a native, evergreen shrub forms thickets up to 7 m tall with increased leaf area index (LAI), increasing community productivity and nitrogen input into the system^34,35^. High productivity of shrubs, along with formation of monospecific thickets, influences carbon assimilation and nutrient input into the system. Evidence suggests *M. cerifera* has been expanding due to decreased exposure to extreme winter temperatures, which impair shrub physiological functioning^6,13,28^. Warmer temperatures in the winter and cooler temperatures in the summer likely enhance shrub survival and productivity. Further, experimental and modeling results demonstrate that a small increase in minimum temperature cause a shift from grassland to *M. cerifera* dominated alternative stable state^6^.

Although mean temperature modification occurs between grass and shrub regime shift, temperature extremes and variance have yet to be quantified across zones including areas in transition. In order to understand the mechanism of shrub encroachment into coastal grassland, our objective was to quantify microclimate variance, community physiology, and community composition in three vegetation zones: grassland, transition areas, and shrub thickets. We hypothesize that shrub encroachment immediately impacts the community (including transition areas) by reducing species diversity, but increases productivity and transpiration. We further hypothesize that shrub canopy creates self-reinforcing microclimate modification. We use a time-series analysis as a novel way to interpret microclimatic modification to better understand the mechanisms of shrub encroachment.

## Results

### Species composition and physiology

Species richness was highest in grassland (6 ± 0) and transition plots (5 ± 0) (Table s.1). Grassland had highest cover of graminoid species (86 ± 2%), which was significantly higher than transition and shrub plots (F = 84.65, *P* < 0.0001, Table 1). Forb cover differed between grass (14 ± 2%) and shrub plots (1 ± 2%) only. Transition areas decreased grass cover (32 ± 2%) with increased shrub cover (61 ± 2%) compared to grassland plots. Shrub plots effectively excluded all other plant functional types, reducing species richness to 2 ± 0 and exhibiting 98 ± 2% woody cover (Table s.1, Table 1). Percent cover for species composition shifted from dominant grass, *Spartina patens*, in grasslands to shrub monoculture of *Morella cerifera* with *Baccharis halimifolia* found along the edge (Table s.1). Coastal communities tend to have low vegetation diversity. Establishment of shrubs reduced Shannon-Weiner diversity from 1.7 ± 0.1 in grassland to 0.1 ± 0.1 in fully thicketized shrubs (F = 68.98 *P* < 0.0001, Table 1). Transition plots did not differ in diversity from grassland.

**Table 1:** Plant functional type % cover using the Daubenmire method and Shannon-Wiener biodiversity across zones on Hog Island, VA. Values represent mean ± SE. Letters represent differences among zones according to the Tukey post-hoc test.

Variable	Grass zone	Transition zone	Shrub zone
Graminoid cover	86.1 ± 2.2^a^	32.4 ± 2.2^b^	0.9 ± 2.2^c^
Forb cover	13.9 ± 2.4^a^	6.7 ± 2.4^ab^	1.0 ± 2.4^b^
Woody cover	−^a^	60.9 ± 2.3^b^	98.1 ± 2.3^c^
H’ Diversity	1.7 ± 0.1^a^	1.6 ± 0.1^a^	0.1 ± 0.1^b^

**Figure 2:**
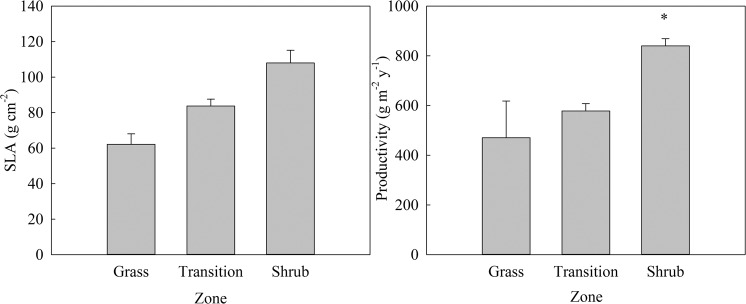
Specific leaf area (SLA; left) and annual net primary productivity (ANPP; right) across the three vegetation zones. Values represent mean ± SE. The asterisk represents significant differences using a post-hoc Tukey Test.

**Figure 3:**
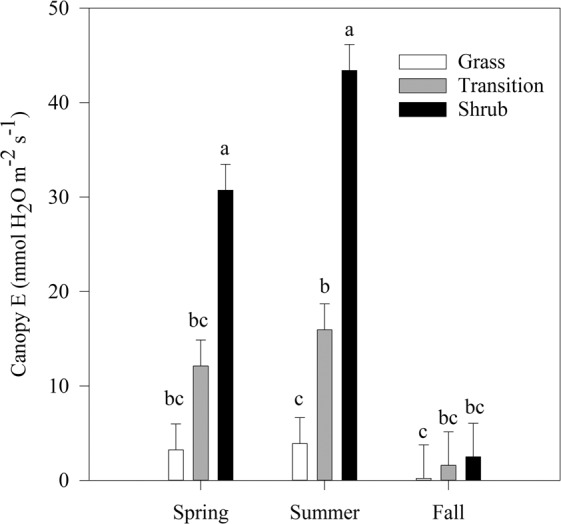
Two-way ANOVA of seasonal transpiration (mmol H_2_O m^−2^ s^−1^) across zones. Values represent mean ± SE. Letters indicate differences among season and zone based on Tukey post-hoc test.

Despite differences in plant functional type and species composition, SLA did not differ among zones (F = 1.80, *P* = 0.208, Fig. 2) although there was a trend towards higher SLA in shrub plots. ANPP was highest in full shrub thickets (*χ* = 9.0, *P* = 0.0111) and 1.4–1.8 x greater than transition and grass plots (Fig. 2). Establishment of shrubs increased transpiration (*E*) during spring and summer (season x zone interaction, F = 9.16, *P* < 0.0001, Fig. 3). In spring, *E* was 2.5–9.5 x higher than transition and grass plots. In summer, shrub *E* was highest, with a 3–11 fold increase relative to transition and grass plots^,^ respectively. Transition *E* trended toward being higher than grass zones by 12 and 14 mg H_2_O m^−2^ s^−1^ in spring and summer respectively but was not statistically different in either season (Fig. 3).

### Microclimate

**Figure 4:**
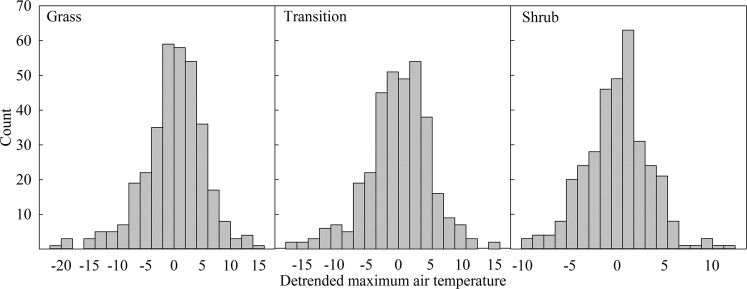
Frequency distribution of detrended maximum air temperature for grass, transition and shrub zones from Hog Island, Virginia.

**Figure 5:**
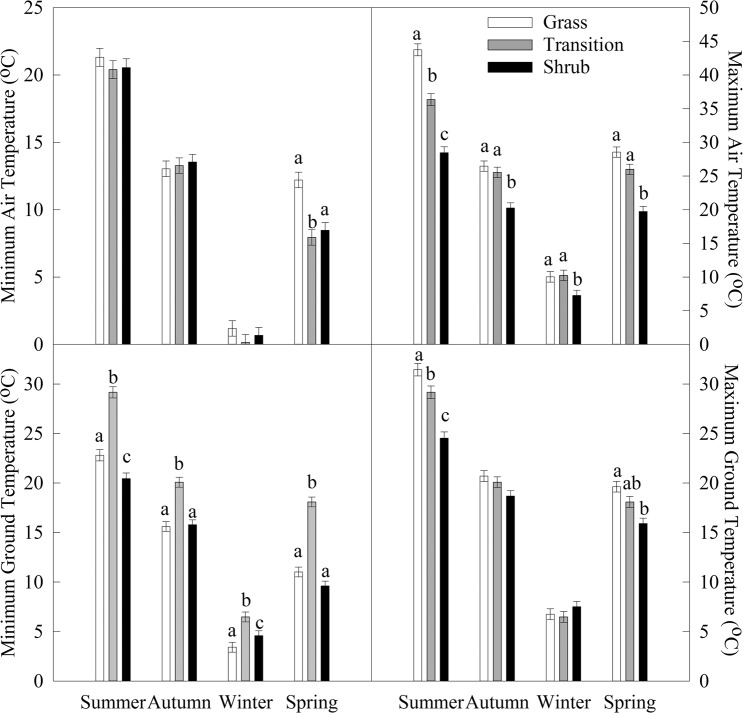
Mean maximum and minimum air and ground temperatures across the grass, transition, and shrub zones divided into season. Bars represent mean ± SE. Letter codes denote significant differences with Tukey test within each season among zones.

Shrub plots exhibited lower variance in both ground and air temperatures relative to grass and transition plots, most notably in in maximum air temperature. Shrub variance was 13.4 °C and 18.2 °C lower than transition and grass, respectively (shrub σ^2^ = 11.9, transition σ^2^ = 25.3, and grass σ^2^ = 30.1) (Fig. 4). Grass and transition plot maximum air temperatures were not normally distributed (K-S = 0.0073, *P* < 0.001 and K-S = 0.056, *P* = 0.013, respectively) where shrub plots were normally distributed (K-S = 0.041, *P* = 0.167). Annual temperatures in grass plots exhibited a wider range and higher variance (Fig. 4). Shrub plots had the lowest mean maximum air temperature throughout all of the seasons (*P* ≤ 0.0125) (Fig. 5). Minimum ground temperatures were altered throughout all seasons as well; transition plots had consistently higher temperatures than shrub and grass. All three vegetation zones were different in summer for maximum air and ground temperature and minimum ground temperature (*P* ≤ 0.0125).

Auto-regressive conditional heteroscedasticity (ARCH) showed that shrub zones exhibited a local effect of variance across maximum and minimum air temperatures as well as maximum ground temperature (*P* = 0.0022, *P* = 0.0021, *P* = 0.0109, respectively; Table 2). Grass zones had a marginal local effect of the temperature variance in minimum ground temperature only (*t* = 2.03, *P* = 0.04). Transition zones had a local effect in minimum air temperature only (*P* = 0.03).

**Table 2:** Autoregressive conditional heteroskedasticity results for maximum and minimum temperatures in grass, transition, and shrub zones on Hog Island, VA. Significant values in bold indicate local control of one day on the next.

Zone	Max air	Min air	Max ground	Min ground
Grass	*t =* 1.04, *P =* 0.2987	*t =* 0.54, *P =* 0.5894	*t =* 0.92, *P =* 0.3570	*t =* 2.03, ***P*** = **0.0425**
Transition	*t =* 0.89, *P =* 0.3733	*t =* 2.24, ***P*** = **0.0254**	*t =* 0.96, *P =* 0.3376	*t =* 2.01, ***P*** = **0.0448**
Shrub	*t =* 2.98, ***P*** = **0.0029**	*t =* 2.30, ***P*** = **0.0217**	*t =* 2.35, ***P*** = **0.0190**	*t =* 1.95, *P =* 0.0511

**Figure 6:**
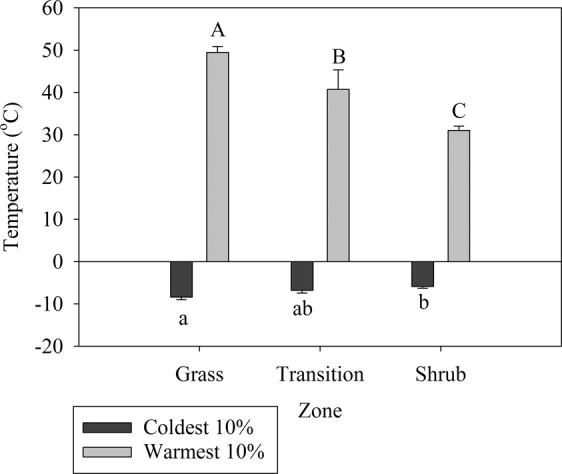
The 10% most extreme temperatures during the winter (black) and summer (white). Values represent mean ± SE. Letters represent statistical differences.

Extreme warm temperatures in summer were higher in grassland plots relative to transition and shrub plots (Fig. 6). Shrub plots were cooler than grass in the summer by 18.4 °C and the warmer in the winter by 2.5 °C (*P* = 0.0013 and *P* = 0.021 respectively, Fig. 6). Winter and summer extreme temperatures did not differ between shrub and transition plots (*P* = 0.058, Fig. 6). Shrub zones exhibited cooler mean and maximum temperatures than transition and grass zones (*P* < 0.001 for both) (Table s.2). Annual mimimum temperatures did not differ statistically (*P* = 0.07) (Table s.2).

## Discussion

Encroachment of *Morella cerifera* in coastal systems has consequences for landscape function by creating new ecological scenarios with increased shrub cover and loss of other species^33^. *Morella cerifera* rapidly expands across the landscape, creating full monospecific thickets within ~15 years after seedlings establish, and arrests succession preventing establishment of maritime forest^36^. We show that shrub presence decreases extreme temperatures and temperature variance, even in transition areas with freestanding shrubs that have not coalesced into thickets. Annual maximum temperature is lower within shrub thickets. The immediate effect of shrub presence impacts evapotranspiration, potentially creating a positive feedback with shrub growth^37^. Once shrubs coalesce into expansive thickets, they reduce biodiversity but increase productivity, a notable departure from expected biodiversity-productivity relationships^38–40^.

Macroclimatic warming has been documented along the VCR over the last 32 years and associated with *M. cerifera* success^6,28^. Here we show that microclimatic temperature moderation reduces summer extreme temperatures with shrub presence, even before coalescence into full thickets (i.e. 8.7 and 18.4 °C cooler in transition and shrub zones, respectively). The transition zone is similar to both grass and shrubs with respect to extreme winter temperatures. This expands on our previous understanding of microclimate modification by highlighting the gradual change in extreme temperatures^3,13,41,42^.

Recent literature has demonstrated the importance of climate variance, as mean values may not fully represent climatic influence^10,43^. Here we document through ARCH analysis that shrubs have a significant impact on temperature variance, creating self-reinforcing microclimate modification observed in both minimum and maximum temperatures. Shrubs also impact transition areas through modifying variance of minimum temperatures. As shrubs exhibit hydraulic failure when temperatures are < −15 °C^6^, reduced temperature variance in winter months may be critical to the success of shrub growth. In this system, water is typically not limiting, especially in cooler, winter months^31^. Variability in temperature and water availability may have a larger role in shrub encroachment globally^9,10^.

With the change from grassland to shrubland, we document 3- to 11-fold higher canopy transpiration (*E*) where shrubs are present (transition and shrub zones) relative to open grassland. This has implications for a system reliant on precipitation input^44–46^. When precipitation rate is low, shrubs access the freshwater lens, reducing groundwater, and potentially decreasing water availability for the surrounding plant communities in summer months, which can alter diversity and function^31,47^. Most notably in summer, shrub presence increases transpiration, which may create a positive feedback wherein water evaporation accelerates evaporative cooling and decreases temperature stress, bringing microclimate closer to photosynthetic temperature optimum of 30 °C^48^. Microclimate modification within shrubs likely benefits vegetation function.

The homogenization of the landscape with *Morella* expansion is contrary to other extreme habitats, where encroaching species with open canopies provide refugia to more sensitive species^49^. In this system, transition zones reflect an amalgam between grass and shrub zones in microclimate measures and plant cover; however, our hypothesis that diversity would be reduced in these zones was not supported, and we did not find evidence of transition areas providing refugia for other species. In landscapes where encroaching shrub density is high and nitrogen is added via nitrogen fixation^34^, a sharp decline in species richness is observed^50,51^. For barrier island systems, the decrease in diversity and homogenization of vegetative communities with fully formed thickets across the landscape has negative consequences by modifying sediment dynamics and reducing island migration in response to sea level rise^33^.

Encroachment and altered PFT cover across the zones results in obvious structural complexity change with taller shrubs and more complex branching relative to grassland^35,37,51^. In many studies a standard functional trait used to assess differences in vegetative community functioning (i.e. SLA) is also observed to differ between woody and herbaceous communities^52^. In this coastal system, SLA was not statistically different among the zones despite the difference in PFT and plant forms (i.e. deciduous herbaceous vs. woody evergreen). There is a trend of lower SLA in grassland and higher SLA in shrubland. *Morella cerifera* has unique functional traits for an evergreen species that are representative of a resource acquisitive species^37,52,53^.

Identifying mechanisms of shrub encroachment in a vulnerable landscape is an important part of understanding the consequences for landscape change. Feedbacks between microclimate and transpiration alter overall landscape function change with the regime shift from grassland to shrubland. We use a novel time-series analysis to show that microclimate modification is self-reinforcing through temperature variance in plots where shrubs are present (i.e. both transition areas with free-standing shrubs and full monospecific thickets). Variance in abiotic niche constraints may play a bigger role in shrub encroachment in many habitats; ARCH may be used to better illuminate the drivers of woody expansion globally. Enhanced microhabitat and high physiological activity of the encroaching shrub likely accelerates expansion of *M. cerifera*, leading to a shrub monoculture. This expansion has consequences for ecosystem function as seen in high ANPP, but also creates new ecological scenarios that alter response of barrier islands to sea-level rise^37^.

## Methods

### Study site

Research was conducted on Hog Island in Northampton County, VA 37°27′ N, 75°40′ W, part of the Virginia Coast Reserve (VCR), a National Science Foundation funded Long-term Ecological Research site owned and run by the Nature Conservancy. Hog Island is ~8 km from the Eastern Shore of Virginia, ~12 km long and undergoes less rollover and sediment erosion than smaller, lower topography islands. Between 1984 and 2016, shrub cover increased from 7% of upland to 29% on Hog Island^33^ (Fig. 1). Shrub encroachment has been documented on many other islands in the VCR over the same timeframe^25,33^. Due to heavy encroachment on Hog Island, we focused on the encroachment zone in the southern portion of the island as all suitable habitat, defined by elevation, has been occupied by shrubs north of our study location^29^^,^^30,54^. In 1984, the study area was composed of grassland, marsh, and sandy soils (i.e. bare) that may contain low vegetative cover. By 1998, the area was fully converted to grassland with no interior marsh. Due to dispersal limitations, shrubs reached the southern portion of the island in the last decade, providing an opportunity to study the encroachment process^30^. Shrubs did not dominate the landscape until the late 1970s to early 1980s and this regime shift has been linked through experiments and models to fewer freezing events through since the 1970s^6,23,25^. Historical records indicate that maritime forest occurred on Hog Island in the early 1900s, however, since the 1980s, succession has been altered by development of tall monospecific shrub thickets of *M. cerifera* and lack of transition to maritime forest^29,36^. Forested plots on other nearby islands have transitioned to dominance by *M. cerifera* (Zinnert, unpublished data). Once established, seedlings grow rapidly and shrub thickets persist on the landscape unless lost due to shoreface erosion from sea-level rise^33,55^.

### Experimental design

**Figure 7:**
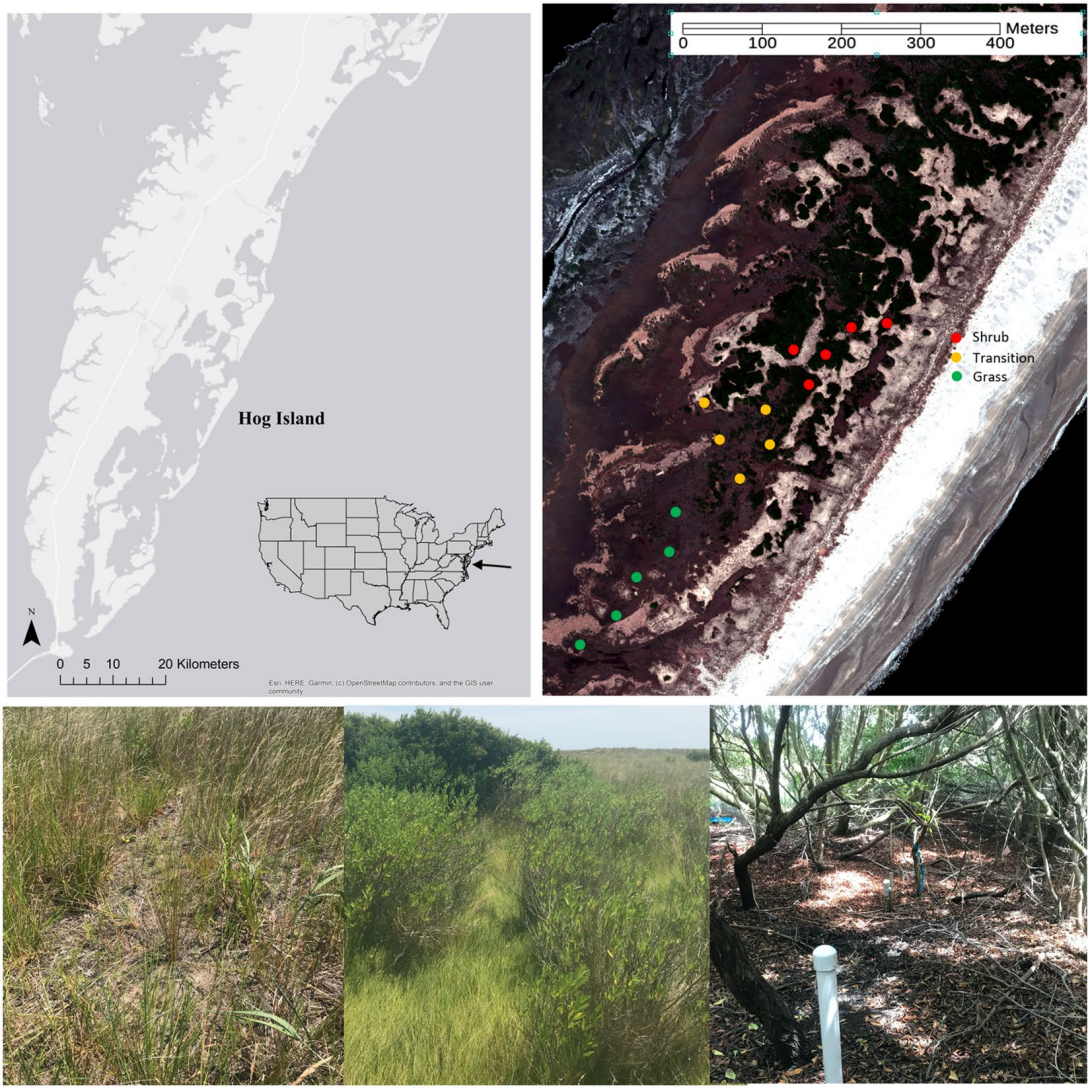
Study location of Hog Island, Virginia. Map produced using © OpenStreetMaps contributors from the ArcGIS basemap, permitted under an open license for academic and commercial use, created by Lauren K. Wood. Approximate plot locations of vegetation types are overlaid on high resolution 2013 imagery^61^. Representative images of grass, transition and shrub zones. Photos provided by Lauren K. Wood.

A map of the VCR was created using ArcGIS version 10 imagery (Esri, Redlands, CA) (Fig. 7). Fifteen spatially disparate 5 × 5 m plots were established during the summer of 2014 and categorized as one of three vegetation zones: grassland, transition, and shrub zones (Fig. 7). Microtopography is variable at small spatial scales and plays an important role in vegetation composition due to access to the freshwater lens^32^. The range of elevation where *M. cerifera* exists is narrow^32^ and grassland plots were placed at similar elevations to those of the transition and shrub plots to limit confounding factors. Further, each plot is considered independent as they are separated by geomorphic features such as interior dunes, relic marsh channeling with high salinity, and other topographic features that exclude *M. cerifera* establishment (Fig. 7). These geomorphic features allow for independence of all variables measured in this study. Each shrub plot was placed within thickets separated by the previously mentioned factors. Grass canopy height was ~ 0.7 m maximum, the canopy in the transition zone was ~1.5 m, and thicket canopy was >4 m in height. Plot elevation ranged from 0.7 m to 1.6 m above sea level. Within each plot soil, surface and air temperature were measured hourly from July 2014 to September 2017 (HOBO U23–003, Onset Inc. Bourne, MA).

Species composition was quantified using the Daubenmire method at the end of the growing season^56^. Species composition was divided into plant functional type (PFT) and percent cover was normalized within each PFT to total cover. Shannon-Wiener Diversity index was calculated using the following equation:

$$H{\prime} =-\mathop{\sum }\limits_{i=1}^{R}{p}_{i}\,\mathrm{ln}\,{p}_{i}$$

where *p* is the proportion of individuals of *i*th species.

Specific leaf area (SLA) of the two most predominant species per plot was assessed in 2016 by getting dry weight of 2 cm^2^ leaf area for woody species and a linear portion of leaf for graminoid species of at least 4 cm long. Community-weighted SLA was calculated using up to the two most abundant species, accounting for species that represent >10% in each plot using the following equation:

$$\mathop{\sum }\limits_{i=1}^{R}{p}_{i}{t}_{i}$$

where *p* is the normalized relative contribution of the species and *t* is the species SLA.

Stomatal conductance (Decagon Devices SC-1, Pullman, WA) was measured on north facing, sun-exposed branches of three to five plants of the two most prevalent species in each zone over three seasons. Stomatal conductance (g_s_) was measured during spring, summer, and autumn of 2017 on the same day between 10:00–14:00 in all plots and restricted to sunny days (PPFD > 1300 μmol m^−2^ s^−1^) to minimize confounding factors. Leaf area index (LAI) (Li-Cor 2200 C) was assessed annually at the end of the 2017 growing season. Leaf-level transpiration (*E*) was derived from g_s_ multiplied by daily vapor pressure deficit. Canopy *E* was calculated using up to the two most abundant species in each plot using the following equation:

$$\mathop{\sum }\limits_{i=1}^{R}{p}_{i}{t}_{i}\times LAI$$

where *p* is the normalized relative contribution of the species and *t* is the leaf-level transpiration, multiplied by LAI to get canopy-wide transpiration^57,58^.

Shrub biomass was collected by randomly selecting and clipping 50 shoots of the current year’s growth around the north side of the shrub crowns. Shoots were then dried at 60 °C for 72 hours. Annual net primary productivity (ANPP) was derived using the allometric equation for juvenile *M. cerifera* shrubs^59^:

$$ANPP=707+(31\ast g\,{shoo}{{t}}^{-1})$$

To estimate ANPP of herbaceous species, aboveground biomass in 10 × 100 cm plots was harvested at peak biomass. Biomass was dried and weighed as described above.

### Statistical analysis

One-way analysis of variance (ANOVA) was used to determine differences in extreme temperature events of the 10% warmest temperatures in the summer and coolest temperatures in the winter, PFT, and SLA. ANPP across vegetation zones was analyzed using non-parametric Kruskal Wallace test due to unequal variances. Mean maximum temperature and canopy transpiration (*E*) were analyzed using two-way ANOVA among seasons and zones. Maximum detrended air temperature was tested for normal distribution using a Kolmogorov-Smirnov (K-S) test. Due to unequal variance and lack of normality, Kruskal Wallace test was used to compare among zones within seasons for maximum, minimum air and ground temperatures. A Bonferonni correction was applied to account for multiple comparisons. To compare across zones, temperature data were detrended to remove seasonal variance. Autoregressive conditional heteroskedasticity (ARCH) test of daily mean temperatures addressed temperature variance relative to local microclimate over time. This test, derived from economics, is a time series analysis to test for variance differences among zones; positive ARCH will exhibit high variance leading to high variance of the next day as described in Seekell *et al*.^60^. This is a novel approach to analyzing microclimate data.

## Supplementary information

Supplementary file.

## Data Availability

Temperature data from this project are available at the Virginia Coast Reserve data portal: Wood, L. and J. Zinnert. 2018. Ground and Air Temperatures on Hog Island, VA in grassland, shrub thicket, and transition, 2014-2018. Virginia Coast Reserve Long-Term Ecological Research Project Data Publication knb-lter-vcr.309.1 (10.6073/pasta/51e468eb007b91a9c2905433eb0eb6d9).
