# Supplementary material for: Decreased temperature variance associated with biotic composition enhances coastal shrub encroachment
Source: Sci Rep. 2020 May 19;10:8210. doi: 10.1038/s41598-020-65161-3 (PMC7237465; doi:10.1038/s41598-020-65161-3)
Supplement: Supplementary file 1 — Supplementary file. [file 41598_2020_65161_MOESM1_ESM.docx]

**Decreased temperature variance associated with biotic composition enhances coastal shrub encroachment**

Lauren K Wood^1,2^, Spencer Hays^3^, Julie C Zinnert^1^*

^1^Department of Biology, Virginia Commonwealth University, 1000 West Cary St. Richmond VA 23225

^2^Integrative Life Sciences Doctoral Program, Virginia Commonwealth University, 1000 West Cary St. Richmond VA 23225

^3^Department of Statistics, Indiana University, 919 E. 10th St. Bloomington IN 47408

*jczinnert@vcu.edu

Keywords: Woody expansion, regime shift, microclimate, diversity, time-series

| Species | | Grass zone | Transition zone | | Shrub zone |
| --- | --- | --- | --- | --- | --- |
| *Morella cerifera* | | 0 ± 0 | 76 ± 6 | | 93 ± 3 |
| *Baccharis halimifolia* | | 0 ± 0 | 4 ± 3 | | 4 ± 3 |
| *Spartina patens* | | 72 ± 6 | 62 ± 8 | | 0 ± 0 |
| *Setaria parviflora* | | 5 ± 3 | 11 ± 7 | | 0 ± 0 |
| *Solidago sempervirens* | | 1 ± 1 | 1 ± 1 | | 0 ± 0 |
| *Ammophila breviligulata* | 4 ± 3 | | 0 ± 0 | 0 ± 0 | |
| *Aster lanceolata* | 2 ± 1 | | 0 ± 0 | 0 ± 0 | |
| *Festuca geniuclate* | 2 ± 0 | | 1 ± 1 | 0 ± 0 | |
| Species richness | 6.6 ± 0.4 | | 5.4 ± 0.4 | 1.6 ± 0.4 | |

Table s.1 Mean ± SE of species percent cover using Daubenmire method across vegetation zones on Hog Island, VA

Table s.2 Mean, maximum, and minimum ± SE of annual air temperature among vegetation zones on Hog Island, VA. A Kruskal-Wallis test was performed among zones, Tukey post-hoc results are indicated by different letters (p<0.05).

| Temperature measure | Grass zone | Transition zone | Shrub zone | H, p value |
| --- | --- | --- | --- | --- |
| Mean | 18.7 ± 0.5 ^a^ | 16.8 ± 0.5 ^a^ | 14.2 ± 0.4 ^b^ | 30.34, *P*<0.001 |
| Max | 26.1 ± 0.8 ^a^ | 23.8 ± 0.6 ^a^ | 18.3 ± 0.5 ^b^ | 62.06, *P*<0.001 |
| Min | 11.3 ± 0.5 ^a^ | 9.8 ± 0.5 ^a^ | 10.2 ± 0.5 ^a^ | 5.4, *P*=0.07 |
